# Understanding the Meaning of Loneliness and Social Engagement for the Workings of a Social Network Intervention Connecting People to Resources and Valued Activities

**DOI:** 10.1111/hex.70111

**Published:** 2024-11-22

**Authors:** Rebecca Band, Anne Rogers

**Affiliations:** ^1^ School of Health Sciences University of Southampton Southampton UK; ^2^ Faculty of Medicine, Health and Life Science, School of Health and Social Care Swansea University Swansea Wales UK

**Keywords:** loneliness, qualitative research, social engagement, social isolation, social networks

## Abstract

**Background:**

Addressing loneliness, which is associated with poor mental and physical health, implicates the need for connectivity to a broad set of situated relationships and activities in the contexts of people's everyday lives. Social engagement has been identified as a relevant psychosocial mechanism mediating health and wellness and is central to addressing loneliness. The aim here is to explore the way in which people identified as lonely conceptualise their experiences of loneliness and social engagement for the purposes of incorporating these into the design and workings of an intervention that allows people to map their social networks and connect them to community‐based valued activities.

**Methods:**

Semi‐structured qualitative interviews were undertaken with 20 participants, aged 21–82 years old (mean age 59.7) nested within a pragmatic, community‐based randomised controlled trial in the north and south of England. Participants had wide‐ranging social network sizes (from 1 to 10 individuals) and reported variable impact of loneliness on their lives.

**Results:**

Loneliness consisted as an absence of intimacy in the face of being surrounded by others, a sense of entrapment and boredom, lacking access to meaningful activities and difficulties in relating to others. The analysis highlighted the role that important relationships have in mediating loneliness. Individual readiness, skills and confidence in forming new connections and engaging with new activities are important barriers that exist in overcoming loneliness. For many, wider socio‐political factors, such as transport provision, availability of resources and costs associated with social engagement are also important barriers which are difficult to overcome.

**Implications:**

Exploring the link between feelings, experiences and meaning of loneliness and the way in which a network intervention can be incorporated offers a focus for mediating the richness and opportunities that arise from locality‐based connections and collective activities in the broader social environment. However, any intervention seeking to address loneliness requires a further focus on both individual and relational factors which might contribute to addressing loneliness and increasing a sense of wellness.

**Patient or Public Contribution:**

The study team worked closely with community partner organisations in all aspects of this research, including, the submission of the funding proposal, development of the study protocol and procedures, recruitment, intervention deployment and assessment of implementation. PPI representatives provided advice on participant materials and interview schedules, and project management throughout and contributed to management and steering committee meetings.

## 1 Introduction

Loneliness constitutes an unwanted emotional state that stems from the ‘perceived discrepancy between desired and actual social relationships’ [1], a lack of social intimacy and deficiency in relationships [2]. It is socially patterned, unequally distributed, with greater prevalence amongst younger and older people, migrants, people with long‐term conditions and ethnic minority groups [3, 4]. Current public policy views high rates of loneliness as a public health problem, and has influenced the growing interest in the implementation of social interventions focusing on improving social skills, enhancing social support and increasing opportunities for social contact [5]. However, loneliness is multifaceted, comprising of inter‐connected social, emotional and existential aspects, which may be experienced differently across the life course and in different settings. The way in which people experience loneliness is likely to shape the way in which people utilise social engagement for accessing and participating in community resources and activities and is relevant for identifying appropriate solutions to alleviate loneliness.

Here, we explore the meaning and experiences of loneliness and its relevance in the context of a social network intervention that supports people to reflect on their existing relationships and link to community‐based ties and valued activities.

### 1.1 The Relational Nature of Loneliness

As a response to social, emotional and environmental triggers, loneliness represents an experience shaped by meaning, social‐cultural norms, interaction and marginality [6, 7]. Changes to social and civic participation are considered reasons for increasing rates of loneliness; especially the reduction of the regularity of friendship and neighbour contact, conversational exchange in routine daily living activities and knowledge and familiarity of those around us [8].

A lack of social cohesion and a sense of belonging is accompanied by a sense of social alienation—a disconnection or distancing from previously held values, practices and relations with social groups in a community.

### 1.2 Social Networks and Loneliness

Social contagion (the propensity for a person to adopt the stance of others) has been seen as a key mechanism in the spread of loneliness [9, 10]. Over time, loneliness establishes norms of negative social reactions resulting in the loss of habitual social contact and may be reinforced by contexts of austerity and marginalisation which predispose individuals to loneliness. Social networks created by interactions with social ties (family, community members, significant others) provide potential strategies for action and have the power to facilitate or restrict access to resources, emotional and instrumental support [11]. Interpersonal ties ‘provide sociability, support, information, a sense of belonging and social identity’ [12, p. 228]. Social capital links social ties with the broader social structure. At an individual level ‘cognitive social capital’ describes the values, attitudes and beliefs producing cooperative behaviour [13] and refers to what people feel—the trust one has in other people and reciprocity between people. ‘Structural social capital’, refers to social action, for example, participation in aspects of civil society [14] such as collective efficacy, or participation in voluntary organisations [15]. Both forms of social capital are relevant for our focus on loneliness. In terms of looking to ameliorate loneliness, social capital resource distribution, reciprocity and trust can facilitate a sense of community belonging [16]. Diversifying connections in one's personal community provides individuals with potential access to new social and material resources and shape individual outcomes, through facilitating access to assets (e.g., information, social autonomy and opportunities). Both ‘strong’ and ‘weak’ ties are associated with lower levels of loneliness [17] but weak ties may have greater potential for addressing social isolation [18, 19]. Interaction with weak ties is more numerous adding to a general feeling of social connectedness and a sense of flourishing [20, 21]. They also combine low levels of contact time, emotional intensity and intimacy, with an inbuilt tendency to reciprocate and bridge to new opportunities [18]. The latter may be especially relevant for people who lack confidants or social support, or those with difficult relationships with family or other ‘strong ties’ [22].

### 1.3 A Social Network Intervention: The Logic of Social Ties and the Experience and Meaning of Loneliness

Opportunities for social participation through community assets linked by networks have the potential to feed into the creation of social network interventions focussed on a reduction of loneliness. The ability to participate in activities is influenced by what is available in local environments and spaces of social encounter. Local organisations and places create opportunities for participation by promoting a sense of belonging, promoting interaction and providing collective activities in terms of sports, cultural and artistic events, routines and hobbies. They also provide ‘relational resources’ which engender and contribute to a sense of identity aspiration, ontological security, mastery, self‐esteem, overall life satisfaction and a sense of collective efficacy. The interaction required to participate in activities of everyday life is linked to affiliation providing opportunities to be with other people. Belonging to a group [23] provides possibilities for the development of personal relationships and increasing and maintaining social and activity levels [24].

In this qualitative study, we were interested in learning about the meanings attached to the everyday experience of loneliness in the context of assessing whether a social network intervention could potentially assist in bridging this gap. We asked questions about social relationships and what kind of support they provided, neighbours and community, meeting new people, spending time alone and about their loneliness and whether this had changed over time.

## 2 Methods

### 2.1 Design

This was a qualitative study nested within a pragmatic, randomised controlled trial comparing participants receiving a social network‐based intervention to address loneliness to a wait‐list control group in the context of local community environments [25]. Ethical approval was received from the University of Southampton and NHS research ethics committees.

### 2.2 The PALS Study

Participants were eligible to take part in the PALS trial if they were over 18 years old and were at risk of loneliness and/or social isolation [25]. Recruitment into the wider trial was conducted pragmatically through community‐based partners located within two regions in the north and south of England: community partners identified and approached potential participants and were also trained to deliver the intervention where feasible.

The intervention is an online tool with two main components. The first allows participants to generate a social network map including relevant ties (which may include friends, family, groups, pets, places or online resources) as a means of empirically observing the ways in which people connect to others [26]. This allows for discussion and attention to be focussed more broadly on their ‘personal community’, taking into account informal social relationships and contact, facilitating conversations about the way in which social ties give or withhold companionship, intimacy, support and engagement in activities through collective efficacy [27]. This process (guided by a trained facilitator) opens up possibilities for individuals to reflect on, reimagine and reconfigure new and existing relationships and network engagement and social participation.

Second, the intervention asks people about their previous, current and potential interests and valued activities and matches up their preferences with local (and online) activities and resources. This provides a means of enabling people to access and incorporate resources and connections relevant to everyday life which may help protect against some of the negative impacts of loneliness and social isolation through a process of initiating positive disruption of cognitions and existing entrapments. Uptake depends on the local availability and accessibility of activities, and whether there is sufficient organisational investment in groups and activities as part of a local political economy [28]. In this way, the intervention moves beyond individual‐level approaches prioritising and targeting the changing of individual behaviour acting as a counter to loneliness, prioritising social connectivity and the contexts, resources, practices, priorities and networks of lonely people.

### 2.3 The Qualitative Study

#### 2.3.1 Recruitment and Interview Procedure

Twenty participants were interviewed, ranging from 21 to 86 years old, with a mean age of 59.7 (17.74) and nine of the participants were male (45%). The initial five participants interviewed were selected based on the field notes of the researchers who conducted baseline quantitative data collection with them as part of the PALS trial; they were lonely and able to reflect on their differing experiences of it. Subsequently, participants were purposively selected based on the density (i.e., size) of their personal community captured in the network generator questionnaire captured at the baseline assessment, in conjunction with responses to the ‘How much does loneliness affect your life?’. We aimed to speak to participants at a range of time points during their 6‐month participation in the trial and to sample broadly to reflect the diverse demographic characteristics of participants included within the wider trial. Four participants were from the control group (20%), with 13 participants recruited from the South of England (65%) to reflect the wider recruitment patterns in the PALS study.

Semi‐structured interview schedules were developed within the wider PALS study team and included experts in sociology and health psychology. Open, inductive questions were used to explore experiences of loneliness especially through life transitions, and the meaning and value of connections and relationships. Initially, interviews were conducted face‐to‐face from November 2019 until March 2020 (where *n* = 11 were conducted over the telephone due to the outbreak of the COVID‐19 pandemic). Informed consent was taken in person or by post.

Interviews were conducted by three researchers involved in data collection for the PALS study. All interviews were audio‐recorded and transcribed verbatim. Field notes were completed after each interview and discussed with the PALS study team. Recruitment was stopped once the researchers agreed that data saturation had been reached {Guest, 2020 #417}.

### 2.4 Analysis

Data collection and analysis were undertaken in parallel to allow for purposive sampling based on researcher insights, team discussions and analytical insights, which facilitated an iterative process of analysis. Inductive thematic analysis methods were used and included line‐by‐line coding and constant comparison [29, 30]. A coding manual was developed and refined, with codes checked against the interview data and discussed within the team to ensure the trustworthiness and authenticity of participant experiences. All data broadly relating to experiences of loneliness, relationships with others and their local environment were analysed.

## 3 Results

Two themes are presented here; the first, describes the everyday experiences of living with loneliness and social isolation and the second, explores relationships with others as an integral part of the experience of loneliness.

### 3.1 The Everyday Experiences of Living With Loneliness and Social Isolation

#### 3.1.1 Loneliness as Entrapment and Boredom

People described loneliness as being a private sadness that happened ‘behind a closed door’. This experience was commonly expressed as an acute sense of dislocation from the outside world coupled with a sense of complete and enduring boredom:

It's your day‐to‐day not being filled the way it should be.

(PT7)

Bored. Bored and isolated.

(PT1)

I wake up and I think, it's what I call a ‘nothing’ day, in inverted commas.

(PT16)

The marginalised, mundane and repetitive nature of being confined to the home was often described;

At the moment I've got four walls. I think I could count all the squares on every wall.

(PT18)

Just seeing something different. I'm not seeing the same walls or the same people all the time.

(PT13)

In response to feelings of boredom and alienation from others, participants reported trying to keep busy. This involved filling time to avoid prolonged periods at home ruminating, and attempting to find a balance between doing what one is able to do in such circumstances and not succumbing to it. This included, for example, working within the parameters of the present situation—even if sometimes that meant only watching the TV or looking out of the window:

No, I need to be busy, honestly if I was sitting in my house, I'd just think in my own thoughts and it's not healthy to do that.

(PT12)

Well, if you're feeling very down then all the things that you're sad about it all comes back doesn't it so then you have to put your mind somewhere else … read or watch something.

(PT11)

And I feel very isolated. I did it this morning, I raised the door, I always do it when I come down, I raise the door and see how the world's doing, what the weather's doing.

(PT16)

In addition, some participants disclosed a preference for face‐to‐face interactions when possible:

There are, there are a number of people now that I've got to know over the past year, you know, just acquaintances, but they're always asking after me.

(PT8)

She's someone that I met through [a friend] actually. She lives up the road from me. She's the one that waved at me (laughs). And you know, because I like photography and she's really into photography, we tend to go out on little trips to … like the other month we went and found kingfishers, so we were, you know, photographing kingfishers.

(PT19)

You can get off your arse and go and sit on a stool outside your front door. Somebody, even if it's only the post‐girl, is bound to say hello to you, you know what I mean…

(PT8)

The benefits of organising face‐to‐face interactions likely extend beyond the interaction itself and alleviate some of the boredom associated with loneliness. Specifically, the organisational aspects involved in arranging and undertaking in‐person interactions provide additional structure and purpose to the day (i.e., getting ready, travel to and from destination), which break up the day in ways which digital interactions may not. Beyond these attempts to add routine where there was none, many described a sense that this was just how things were, and that to cope, they must just accept the situation and keep going:

Well I am, because I have to, I don't have any choice. What can I do? Sit there and cry? I can't do—well, I could but that's not going to do me any good. I just have to keep pushing on.

(PT17)

No, I just think must do better. Get up and do something… Yeah, try and be positive I always think. Because it's, you know, a terrible world in some ways isn't it so…

(PT15)

### 3.2 Engaging With Meaningful Social Activity as a Way out of the Imprisonment of Loneliness

Feelings of being socially isolated have been linked not only to an absence of meaningful relationships but also to activity [31], and the results here also highlighted that in line with the boredom experienced, largely, valued activities were also missing. In this respect, opportunities for accessing meaningful social activities were viewed as a potential, if challenging, route to participation as a way of ameliorating loneliness by respondents:

And so actually doing new groups, which is something that I think I ought to do and that I should do and it would be interesting, actually doing it for the first time I would really struggle with, even though I want to do it.

(PT19)

Just to get us open and give the men who are struggling a little bit of light, somewhere they can go where they can have a coffee, something to eat, have a game of snooker…

(PT4)

Readiness in terms of levels of felt stigma, personal self‐worth, and potential social rejection inhibit the ability to think about linking to new activities. In turn, this is linked to a sense of legitimacy of whether one is being judged as being of the appropriate social status, at the right point in the life course to be morally worthy of being lonely and engaging with community activities in particular social situations and settings:

I think maybe there's a stigma attached to loneliness when you're not elderly. I think people think, ‘Well why is she lonely?’ and the example of a mother with a new baby that I used earlier, people probably think, ‘Well why are they lonely?’ I think it's acceptable to be old and lonely but not quite so acceptable to be younger and lonely.

(PT17)

You can't go to anything that's mainly families and children. You can't go there (parent and child groups) on your own because you're going to be judged. So I think for the man, it's a big hurdle to cross, obviously if you've got a girlfriend or a wife that you're going with, you both blend together, but you just sort of stick out like a sore finger, you know.

(PT3)

This fed into a sense of apprehensiveness and wariness about engagement or participation with groups and activities, and the nagging awareness of this impacting on potential for relating to others in everyday encounters. Others lacked the necessary skills and confidence to attempt ameliorative steps:

So yeah, it's a bit, like, embarrassing, humiliating, just, like, you feel a bit like a lost puppy.

(PT4)

Yes, to walk in somewhere on your own is really hard.

(PT15)

I won't show up sort of unannounced on my own because I'm just a little bit shy even though I'm probably, you know, I will talk to anyone and people think that I'm extrovert, I'm really not so it would have been nice to have like a buddy that could come along, introduce themselves, spend several sessions with you, you get used to them and then go along to something that might have been designated suitable for you.

(PT9)

A number of structural barriers were identified as preventing engagement. Being able to physically travel to a community location was seen as an insurmountable obstacle for some, for reasons such as ill health or poor mobility, lack of access to affordable and accessible transport or simply a lack of appropriate activities or services:

Transport and lack of energy and lack of funds and, yeah, because funding for me would be a big thing.

(PT9)

Well, I can't afford it [to go to groups], I'm on Universal Credit. I don't even have my heating on, I can't afford my heating so to fork out for taxis when I can't put my heating on seems a little bit over the top.

(PT17)

It is frustrating because physically I would like to be able to do more but physically I can't! It is so restrictive and it can get depressing at times, but I have managed that pretty well, to be fair.

(PT11)

Yeah, I would like to get out, yeah, but I can't go out alone anymore, I have to accept that.

(PT18)

These structural factors were often seen as things beyond individual control, with little power to overcome and change. Given the potential benefit from engaging in valued activities, there are potential structural and societal missed opportunities for addressing loneliness which are likely to disproportionally affect those who are already most marginalised through lack of accessibility.

### 3.3 Relationships With Others as an Integral Part of the Experience of Loneliness

Several aspects of loneliness linked appraisal of, and feelings about, who they had contact with on a daily basis.

#### 3.3.1 Helping and Being Helped: Mismatched Expectations

Many respondents did not complain about a lack of people around them. They therefore felt lonely even when, or despite being surrounded by others, highlighting the distinction between feelings of loneliness and isolation. Many participants described the ‘lifeline’ that key network members represented in their lives; although this was largely in relation to others being available to provide practical help and support:

Literally she does a hell of a lot for me; she does the laundry, she does my shopping, sometimes she's cooked me dinner. I'm very grateful. Very grateful. Because I can't do a lot, you know?

(PT18)

Ah, well she's very good, she comes, she phones me twice a week and she ferries me to hospital appointments and dentists and things like that.

(PT16)

However, there was an underlying rejection of interdependence and difficulties linked to emotionally opening up and asking others for help. Participants reported not being able to admit to needing help even though they recognised this would be helpful:

I think it's a dual‐edged sword, because there's times when life would be a damn sight easier just for ten minutes if I asked somebody to help me, but my stubbornness won't let me. And apparently it's something I'm well known for.

(PT8)

Yeah, I'm not one for putting on people. You know, I'm sort of self‐reliant even though I can't do actually do very much for myself, but you know what I mean. I know I can't do it … but in my brain, I says I can.

(PT18)

As a result relationships could be strained when the balance of reciprocity felt uneven and participants felt like they were not able to meet others as an equal and offer perceived value to the relationship, and as such led to feelings of guilt or being a burden on others.

Well I'm not giving them company, I'm not, am I? I'm isolated here. The only time I was company was when I was out with them. When they come here, they're company for me, I'm not company for them.

(PT10)

#### 3.3.2 Feeling Alone in the Face of Others: An Absence of Intimacy

The complexities of being able to establish and maintain meaningful relationships with others acts as a barrier to engagement for many. Some described their loneliness in the context of withdrawal from or disconnection from others. Participants reflected on experiencing difficulties in forming and sustaining relationships with others over long periods, often since childhood. Many reported current difficult relationships with close family members and deliberately cutting off from relating to others.

I think I sort of cut my nose off to spite my face… I have sort of cut myself off from a lot of things…

(PT2)

…I still go through little periods where I don't want to know anyone or anything; whether that's life or not I don't know.

(PT13)

I don't particularly want to interact too much, it's just anxiety as well, you know, if you go to somewhere new, you know, it's that first hurdle of you know that it's coming and you've got to do it and then come the morning you think no, I'd rather not, I can't be bothered.

(PT17)

Difficulties forming meaningful relationships were attributed to feelings of being on a ‘different wavelength’ and not fitting in with others in the immediate social context. This was framed as a disconnect between themselves and their connections, specifically relating to how they perceived themselves compared to the people (and consequently the potential for social connections) around them.

… I feel about my neighbours like, I feel like a thoroughbred racing horse in a field of donkeys.

(PT1)

[loneliness is] Not knowing where you fit in.

(PT3)

I sometimes struggle—well, a lot of the time I struggle trying to find common ground.

(PT13)

I sometimes feel a bit hollow inside, when I look at my relationships/friendships, circle of friends. They're a bit … there's something lacking really.

(PT1)

Yeah, it's definitely to do with that [not fitting in]. I can't bear superficial. I get irritated by it.

(PT19)

However, the lack of emotional connectedness meant opportunities for meaningful social interaction with others became limited despite physical connection, proximity to others and the availability of instrumental support. This emotional distance, the inability to connect and an absence of communication presented as a source of hidden distress and anomie:

you can be in a room with a thousand people and feel lonely, I know that for a fact, you know… it's about your mental state and how you handle all the different interactions and things, you know, and how much you worry about how you're perceived and all that sort of thing, you know.

(PT8)

And sometimes I can be in a crowded room and feel lonely and it's a strange one that it's almost like you feel you can't connect, particularly if you're in a room with people that you don't know.

(PT19)

….having no‐one to talk to sometimes within the house, within the household, it means even when everybody's around me I can still be lonely. It means boredom, it means anxiety, it means depression, it means wanting to hide away and become more and more isolated, not wanting to go out because I don't want to interact so it's everything.

(PT9)

#### 3.3.3 Navigating the Social World

Participants described the ways in which they lack the necessary skills, confidence or readiness to build relationships with others:

Sometimes it's breaking the ice and sometimes I need them to break the ice. So, if it's a new person it's quite difficult. Fear of saying the wrong thing or doing the wrong thing.

(PT13)

Like the girl from work invites me out with them and I make all sorts of excuses not to go. Then I say, ‘Right, I am going to go, it's my own fault, I am going to go’, and then when I get to the day I think, ‘Oh I can't go’.

(PT19)

*…* I don't see me forming friendships. It's been so long since I've formed a friendship other than at work, I don't see me doing it.

(PT17)

Obviously the nervousness, the anxiety, and … there's also this thing like I hope they like me, you know what I mean? And that I hope I'm good enough, you know?

(PT9)

Past experiences, insecurities and lack of skills contributed to wariness of ‘new’ others and their intentions. When trust is broken, or an element of mistrust comes in to the relationship it will prevent new ties being formed:

Yes, it [mistrust] holds me back from approaching people, a lot, or trying to become friendly. It holds me back from getting to know, trying to get to know people.

(PT1)

I think that's what the problem is; I think it's, like, loneliness and being very careful who you become friends with, because a lot of people…they're not worth having as friends.

(PT3)

The unavailability of emotional support or companionship from one's immediate network required participants to look for connectivity further afield as a way of managing this. The use of the internet and social media opened up opportunities to connect with people and groups around shared interests, as well as provide a means for communicating with others outside of the immediate physical environment:

Yeah, I've recently joined … my favourite band of all time was Madness. And I've recently joined the Madness fan group thing online. And then this guy asked me to be his friend and then he asked me to join all his other groups that he runs. So, in that sense, that's quite good to sort of join in that. What else? Cats. I've got a cat lovers' group. And black cats are the best sort of thing, because mine's a black cat. So, yeah, that's quite nice.

(PT19)

I mean, it's what I call cyber social life, yes. Yes, I get enough emotional strength, I get enough emotional nourishment, if you know what I mean.

(PT1)

I interact with a lot of people online.

(PT11)

This was especially useful for individuals who were limited physically, for example, by illness or having small children, which made the possibility of finding new, meaningful connections in‐person challenging. However, for others, using digital spaces to interact was something that they weren't interested in engaging with ‘I don't understand this going online thing’ (PT6).

## 4 Discussion

This study explored the way in which meanings people ascribe to the experience of loneliness and social isolation and what is done to address this in everyday life. The results illuminate how the experience and meaning of loneliness is one of being grounded in alienation from existing social connections and the relevance of engaging activities as a potential mechanism to alleviate loneliness. Both personal communities and connections to valued activities are mechanisms to manage the distress associated with social isolation. Both offer the potential to be employed or accessed both digitally or in‐person and importantly, intersect with marginalisation.

Our qualitative analysis highlighted the way in which the meanings people ascribe to the experience of loneliness and social isolation and what they do to address this in everyday life are linked to attempts at amelioration through social strategies. A key element of the PALS study is engendering a readiness to engage with community activities through reflecting on existing connections and activities and thinking through how this could be changed for the future. The extent to which these strategies may be successful depends on factors related to the individual, the network and the wider social structures within which these are situated. Whilst individuals within our study scored highly on loneliness and provided detailed descriptions of their daily lived experience, often feeling lonely was not simply a result of being alone but also of an underlying inability to relate to others. The analysis presented here highlighted that for these individuals, the meaning of loneliness consists of an absence of intimacy in the face of being surrounded by others, a sense of entrapment and boredom combined often with having limited access to meaningful activities or resources to address their sense of loneliness or isolation.

The need for individuals to connect with different people in, and beyond, their immediate networks depending on their specific needs has been examined elsewhere [32]. Previous research has highlighted the specific network processes involved in harnessing personal community support, which involve a process of first being able to identity who is the most appropriate person within their network before they are able to ask for help or support [27, 33]. The presence of efficacy within the network alone is insufficient if individuals are unaware or unable to access it to meet their needs. In this case, and in contrast to previous work on the role of social networks in long‐term condition management where practical support is utilised [19], the individuals interviewed here expressed lacking the emotional connections and ability to engage with support from those around them. Having people in close proximity was insufficient to alleviate feelings of loneliness, and in fact, may increase feelings of alienation when there is an absence of belonging with one's personal community. It is also possible that those people who become lonely enough to be identified for inclusion in a trial like the one in the current PALS study are situated within ‘lonely networks’ where physical connection but emotional disconnection and shared environments have facilitated the spread of loneliness [9, 10]. Additionally, entrenched aspects of the way in which relationships and relationality are experienced by those who are lonely (as opposed to simply being social isolated) may block the ability to engage. In this respect, future interventions designed to address loneliness may require the addition of more opportunities for personalised work in addition to reflecting on networks. Specifically, it may require intervening to address psychological aspects of loneliness (such as cognitions about the social self and others, and building confidence and efficacy in social situations) may need to be addressed as a precursor to engagement with others and the wider community [34].

The online network mapping platform used within the study allows for the positive disruption of loneliness through the visualisation of a personal community, and discussion of network resource engagement with reference to new or renewed support or activities. This exploration offers the possibility to understand how people may access others with shared interests. While the internet might in itself act as an activity to distract from the painfulness of loneliness [35] online platforms provide one possible way of diversifying the availability of resources and overcoming locality restrictions where possible to access like‐minded people (notwithstanding the digital exclusion of some social groups in the population and recognising the need for access to the internet and technology in doing so). However, for those who are chronically isolated and lonely, locally‐based and physical interactions offer great potential benefits for addressing the feelings of boredom and entrapment as well as increasing cognitive forms of social capital such as trust and norms of reciprocity.

There is a strong connection between poverty and the risk of social isolation [36] and evidence suggests that locality‐based connections and activities have to be very local and proximate for those who are marginalised and lack resources to be able to link to them [37]. In some cases, the lack of personal resources are a significant barrier that stop people engaging; social and structural resources such as finance, transport, mobility are required to engage with social and local opportunities. Thus, the likely traction, uptake and sustainability of interventions that seek to harness the power of social capital and community resources need to be seen in the context of the availability and sustainability of community assets. This suggests that investment in community assets and resources (including things such as the availability of affordable and reliable transport) is essential in providing opportunities for those who are isolated or lonely.

### 4.1 Strengths and Limitations

The majority of participants interviewed in this study were from the intervention group; the potential that this may have biased accounts or experiences of loneliness and therefore skewed the results presented here must be acknowledged. However, the analysis of the larger demographics of the PALS RCT highlights that there were no significant demographic differences between the control and intervention group participants who were randomly allocated on the same criteria to each arm of the trial.

## 5 Conclusion

Taking a social network‐informed approach to address loneliness enhanced understanding of mechanisms to address loneliness by bringing together the experience of loneliness and how it is allocated meaning in the context of valued relationships and activities within people's daily lives. Alienation or disconnection from existing social ties can be deleterious, fostering and reinforcing a sense of entrapment and low self‐worth whilst a social intervention designed to foster new connections and activities holds out the hope of countering this. However, our findings here suggest that future interventions should include the psychological components outlined above, where appropriate, and that strategic investment in local, community‐based assets and services would be beneficial at addressing the systemic elements of loneliness which affect those who are most vulnerable.

## Author Contributions

**Rebecca Band:** conceptualisation, funding acquisition, writing–original draft, writing–review and editing, formal analysis, investigation. **Anne Rogers:** conceptualisation, investigation, funding acquisition, writing–original draft, writing–review and editing, formal analysis.

## Conflicts of Interest

The authors declare no conflicts of interest.

## Data Availability

The data that support the findings of this study are available from the corresponding author upon reasonable request.
